# Supplementary figures and images for: Complex spatio-temporal distribution and genomic ancestry of mitochondrial DNA haplogroups in 24,216 Danes
Source: PLoS One. 2018 Dec 13;13(12):e0208829. doi: 10.1371/journal.pone.0208829 (PMC6292624; doi:10.1371/journal.pone.0208829)

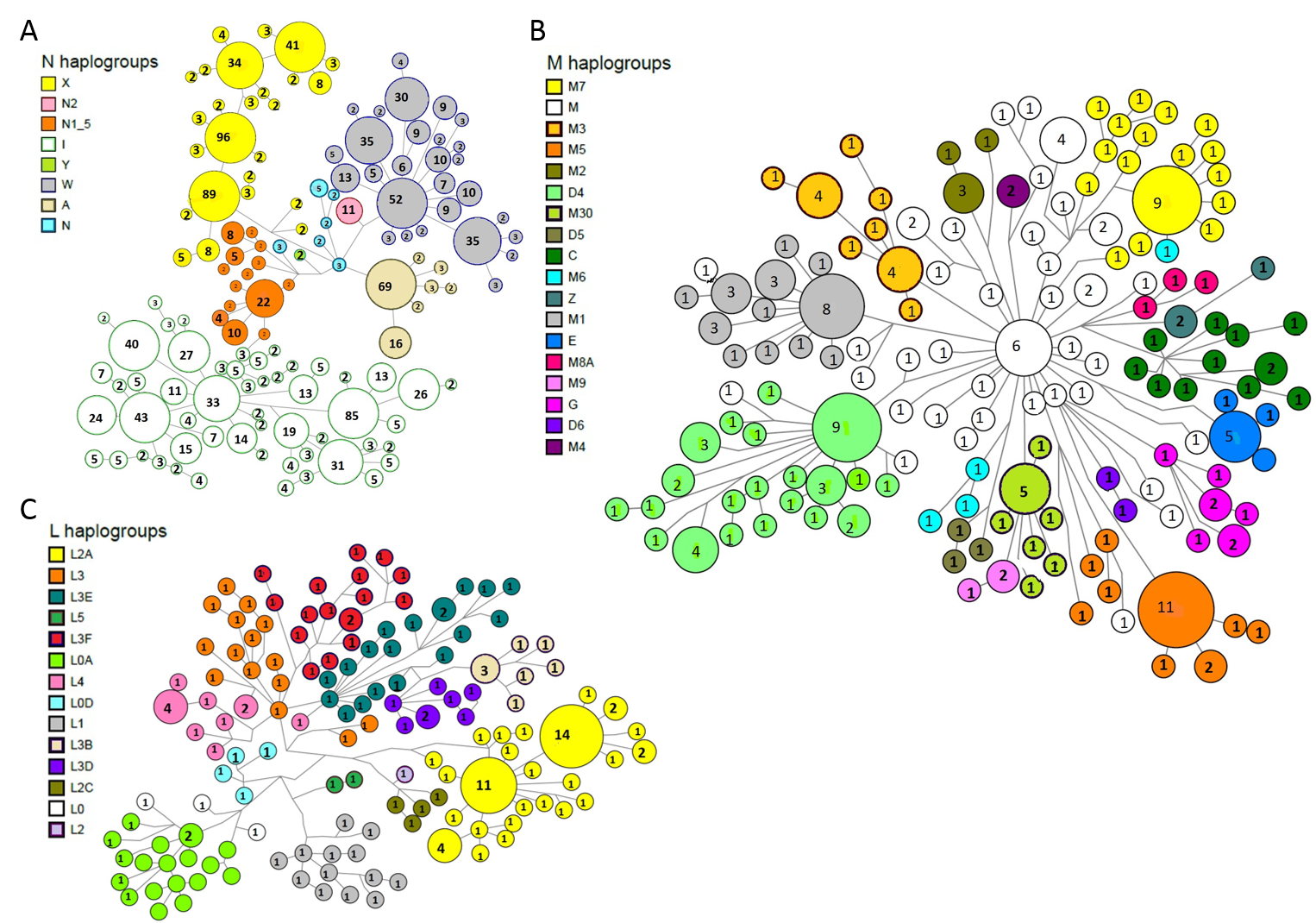

Supplement: S1 Fig — M-J Network of haplogroups belonging to A) N-macrohaplogroup, B) M macrohaplogroup and C) L macrohaplogroup. (TIF) [file pone.0208829.s001.tif]

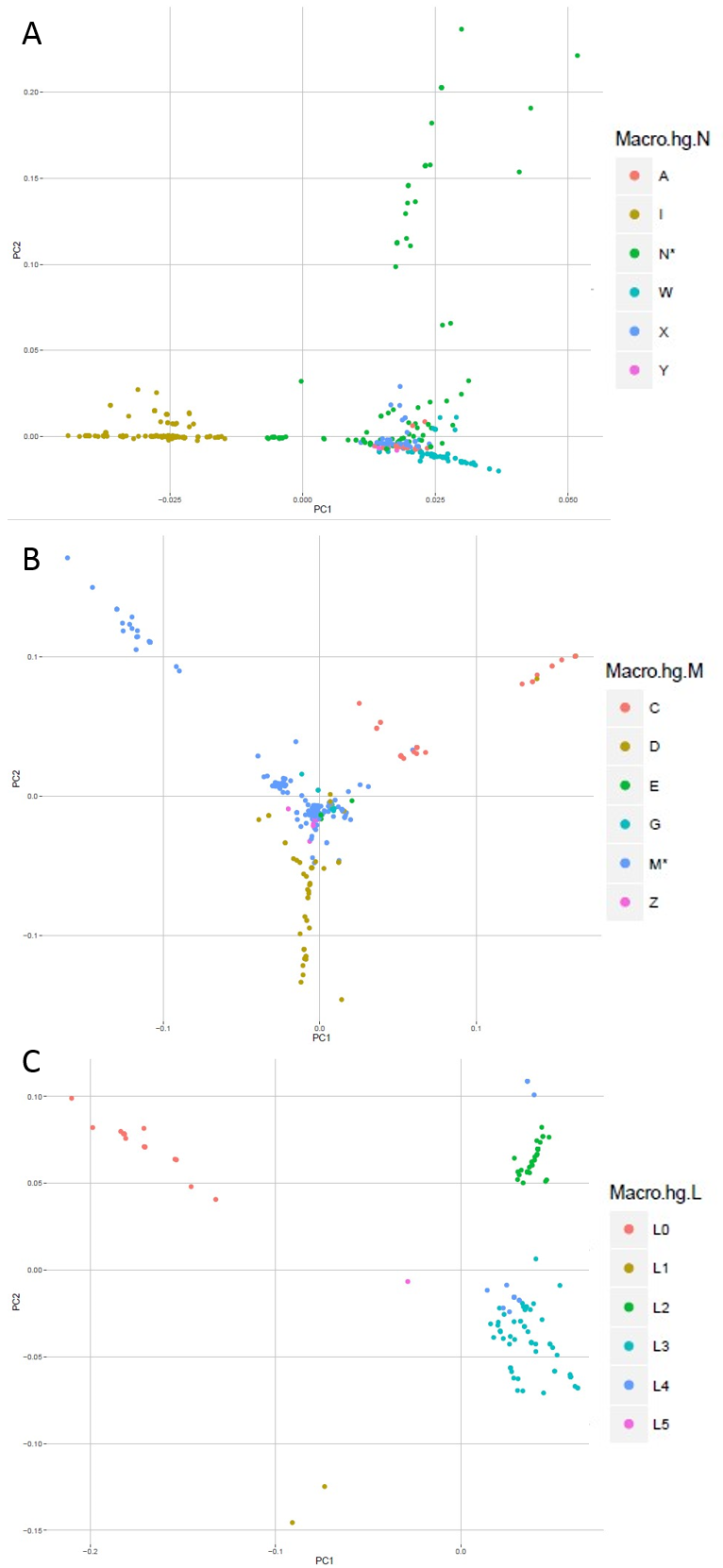

Supplement: S2 Fig — PCA of mtDNA SNPs from persons belonging to A) N macrohaplogroup, B) M macrohaplogroup and C) L macrohaplogroup. The affiliation to haplogroup is shown with color coding. (TIF) [file pone.0208829.s002.tif]

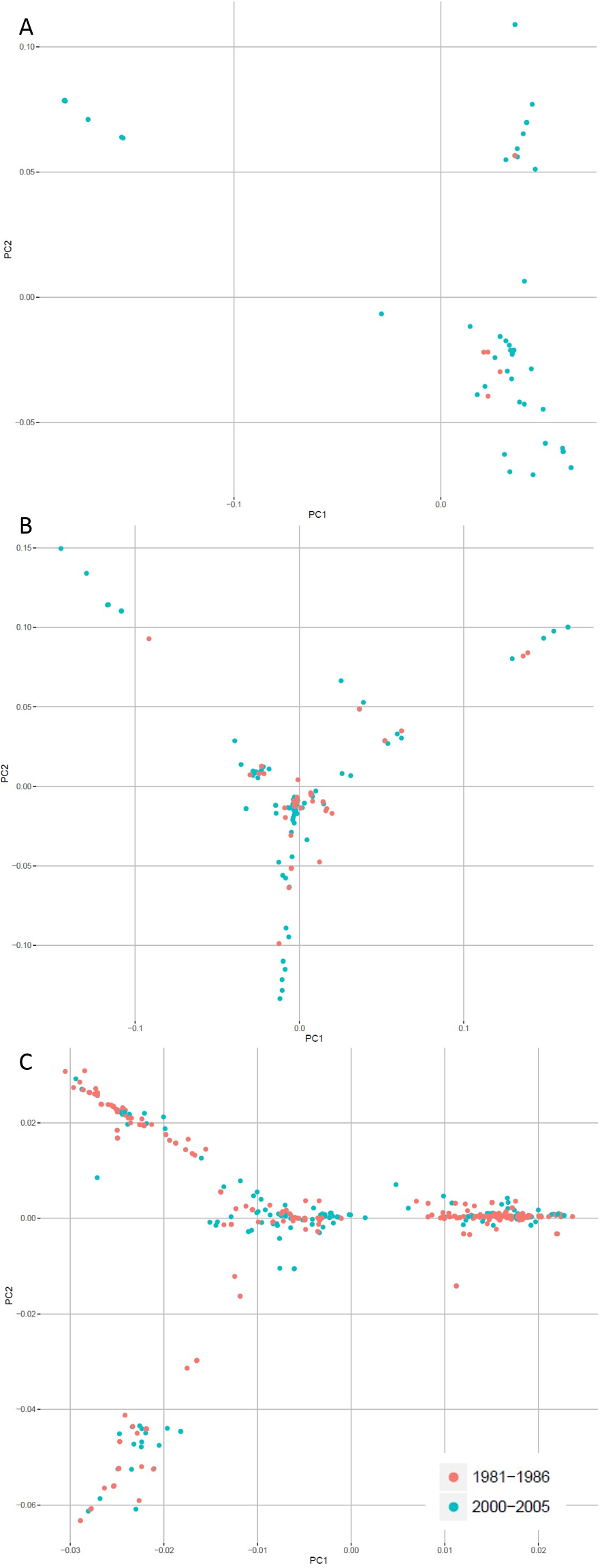

Supplement: S3 Fig — PCAs of A) hg L, B) hg M and C) hg U in 1981–1986 and 2000–2005, PC1: First principal component. PC2: Second principal component. (TIF) [file pone.0208829.s003.tif]

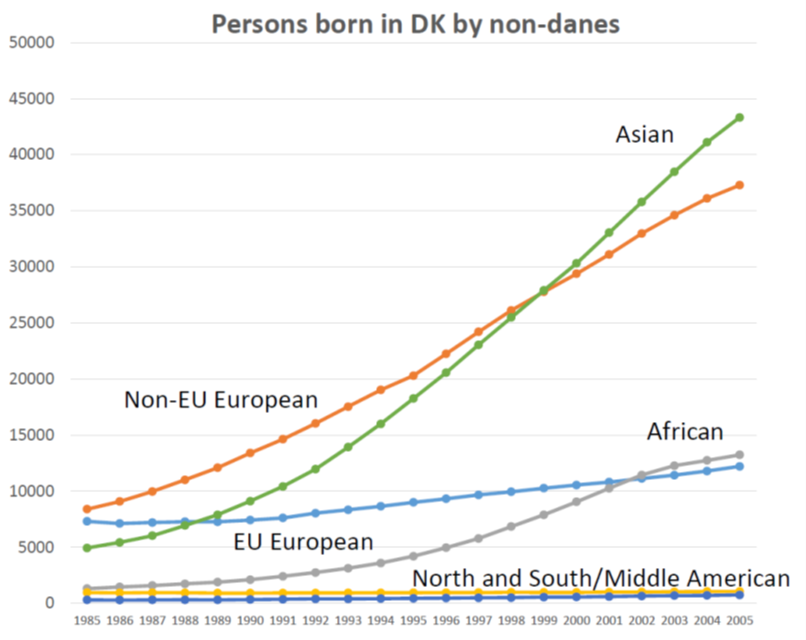

Supplement: S4 Fig — (TIF) [file pone.0208829.s004.tif]

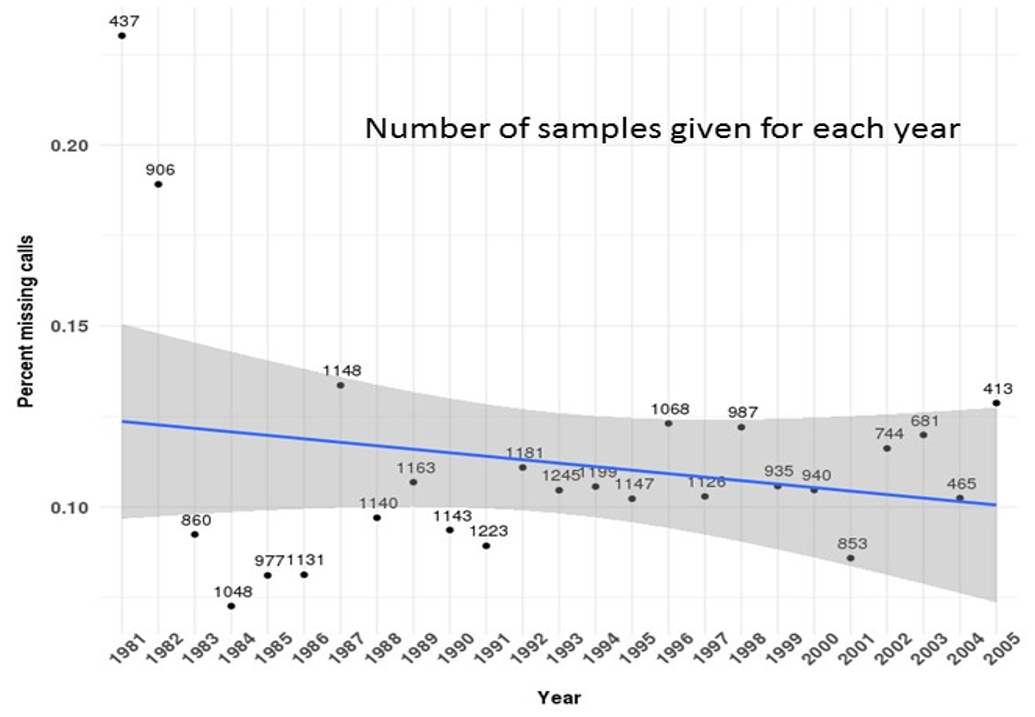

Supplement: S5 Fig — (TIF) [file pone.0208829.s005.tif]
