# Supplementary material for: Complex spatio-temporal distribution and genomic ancestry of mitochondrial DNA haplogroups in 24,216 Danes
Source: PLoS One. 2018 Dec 13;13(12):e0208829. doi: 10.1371/journal.pone.0208829 (PMC6292624; doi:10.1371/journal.pone.0208829)
Supplement: S1 Table — (DOCX) [file pone.0208829.s006.docx]

**S1 Table.** Haplogroup and sub-haplogroup defining SNPs and call frequency

| Macro-haplogroups: L0,L1,L2,L3,L4,L5,L6,M*D,N*,R* | | | |
| --- | --- | --- | --- |
| ProbeID | rCRS position | Null_call % | Diagnostic for haplogroup (variant): |
| exm2216204 | 1048 | 0,0 | L0 (T) |
| MitoG10590A | 10589 | 0,0 | L0 (A) |
| MitoG3667A | 3666 | 0,0 | L1 (A) |
| MitoA7056G | 7055 | 0,0 | L1 (G) |
| exm2216244 | 2416 | 0,0 | L2 (C.) |
| exm-rs41378955 | 16390 | 0,3 | L2 (A) |
| exm-rs3021088 | 5460 | 0,0 | L4 (A), L3 (G) |
| MitoG15044A | 15043 | 0,0 | L4 (G), L5 (G), L3(G),M*D (A) |
| exm2216421 | 12950 | 0,0 | L5 (G) |
| MitoC16149T | 16148 | 0,5 | L5 (T) |
| MitoC9541T | 9540 | 0,0 | L4 (C), L3(C), M*D (C), N*(T) |
| MitoA15302G | 15301 | 0,1 | L4 (A), N*(G) |
| exm2216190 | 709 | 0,2 | L6 (A) |
| exm2216201 | 961 | 0,0 | L6 (C.) |
| MitoC13651T | 13650 | 0,0 | L3 (C.) |
| MitoT12706C | 12705 | 0,6 | N*(T), R*(C.) |
| Cumulative NULL_call %: | | 1,9 |  |
| Macro-haplogroup M*D | | | |
| MitoG15044A | 15043 | 0,0 | M*D(A) |
| MitoC9541T | 9540 | 0,0 | M*D(C.) |
| Cumulative NULL_call %: | | 0,0 |  |
| exm2216447 | 14318 | 0,0 | C(C), Q(T) |
| MitoT15785C | 15784 | 0,0 | Z(C.) |
| exm2216257 | 3027 | 0,0 | E (C.) |
| exm2216420 | 12940 | 0,0 | Q(A) |
| exm2216340 | 8414 | 0,0 | D4(T), D6(C.) |
| MitoG3916A | 3915 | 0,0 | Q(G) |
| 200610-108 | 1107 | 0,5 | D5(C.) |
| exm2216190 | 709 | 0,5 | G(A) |
| 200610-120 | 5108 | 0,0 | G(C.) |
| 200610-6 | 4833 | 0,5 | G(G) |
| 200610-59 | 9785 | 0,0 | D3(T) |
| Cumulative NULL_call %: | | 1,5 |  |
| Frequent haplogroups. | | | |
| MitoG15044A | 15043 | 0,0 | L(G), M*D(A) |
| MitoC9541T | 9540 | 0,0 | L(C.), M*D(C.), W(T), I(T), C(T), N*O,S,Y,A(T) |
| MitoT12706C | 12705 | 0,6 | R-subhaplogroups (C.), N-subhaplogroups(T) |
| exm-rs2853498 | 12308 | 0,0 | U(G), K(G), R*HV,B,P(A) |
| MitoC295T | 295 | 0,0 | J(T) |
| MitoA11252G | 11251 | 0,2 | J(G), T(G), R*HV,B,P(A) |
| exm2263337 | 15928 | 0,3 | T(A) |
| exm2216232 | 1888 | 0,1 | T(A) |
| MitoA11468G | 11467 | 0,1 | U(G), K(G) |
| MitoA10551G | 10550 | 0,0 | K(G), U(A) |
| exm-rs2015062 | 7028 | 0,3 | H(C),R*HV,B,P(T) |
| exm2216249 | 2706 | 0,1 | H(A) |
| exm2263341 | 15904 | 0,0 | V(T), R*HV,B,P(C.) |
| exm2216208 | 1243 | 0,1 | W(C), N*O,S,Y,A (T) |
| exm2263322 | 10034 | 0,0 | I(C), N*O,S,Y,A(T) |
| MitoT6222C | 6221 | 0,1 | X(C), N*O,S,Y,A(T) |
| exm2263329 | 13966 | 0,7 | X(G) |
| exm-rs3021088 | 5460 | 0,0 | W(A) |
| Cumulative NULL_call %: | | 2,7 |  |
| Macro-haplogroup N* | | | |
| MitoT12706C | 12705 | 0,6 | N*(T) |
| MitoC9541T | 9540 | 0,0 | N*(T) |
| Cumulative NULL_call %: | | 0,6 |  |
| exm2216208 | 1243 | 1,0 | W(C.),N*(T) |
| exm2263322 | 10034 | 0,0 | I(C.), N*(T) |
| MitoA1738G | 1736 | 0,1 | A(G), N*(A) |
| MitoT6222C | 6221 | 0,3 | X(C.), N*(T) |
| exm2263329 | 13966 | 0,1 | X(G) |
| exm-rs3021088 | 5460 | 0,1 | W(A) |
| 200610-129 | 8404 | 0,1 | S(C.) |
| exm2216444 | 14178 | 0,0 | Y(C.) |
| Cumulative NULL_call %: | | 1,7 |  |
| Macro-haplogroup R* | | | |
| MitoT12706C | 12705 | 0,6 | R*(C.) |
| Cumulative NULL_call %: | | 0,6 |  |
| MitoC295T | 295 | 0,0 | J(T) |
| MitoA11252G | 11251 | 0,2 | J(G), T(G), R*HV,P(A) |
| exm2263337 | 15928 | 0,2 | T(A) |
| exm2216232 | 1888 | 0,1 | T(A) |
| MitoA11468G | 11467 | 0,2 | U(G), K(G) |
| exm-rs2853498 | 12308 | 0,0 | U(G), K(G),R*HV+B+P(A) |
| MitoA10551G | 10550 | 0,0 | K(G), U(A) |
| exm-rs2015062 | 7028 | 0,3 | H(C),R*HV,P(T) |
| exm2216249 | 2706 | 0,0 | H(A) |
| MitoG10311A | 10310 | 0,0 | F(A), R*HV,P(G) |
| exm2263341 | 15904 | 0,0 | V(T), R*HV,P(C.) |
| exm2216269 | 3497 | 0,1 | F(C.) |
| exm2216421 | 12950 | 0,0 | B(G) |
| 200610-61 | 11061 | 0,0 | B(T) |
| MitoT9951C | 9950 | 0,0 | B(C.) |
| 200610-146 | 16217 | 0,3 | B(A) |
| Cumulative NULL_call %: | | 1,6 |  |
