# Supplementary material for: Complex spatio-temporal distribution and genomic ancestry of mitochondrial DNA haplogroups in 24,216 Danes
Source: PLoS One. 2018 Dec 13;13(12):e0208829. doi: 10.1371/journal.pone.0208829 (PMC6292624; doi:10.1371/journal.pone.0208829)
Supplement: S2 Table — In brackets are given the tables that are referred to in the manuscript. (DOCX) [file pone.0208829.s007.docx]

| Macro-hg and hgs | n | % |
| --- | --- | --- |
| L0-L6, M, N, R - macrohaplotyping (Table 1) | 426 | 1.7 |
| N-haplotyping (Table 4) | 30 | 1.8 |
| M-haplotyping (Table 4) | 20 | 5.1 |
| R-haplotyping (Table 2) | 104 | 0.5 |
| -H-subhaplotyping (Table 3) | 129 | 1.2 |
| -U-subhaplotyping (Table 3) | 65 | 2.0 |
| -K-subhaplotyping (Table 3) | 60 | 3.4 |
| -J-subhaplotyping (Table 3) | 100 | 4.6 |
| -T-subhaplotyping (table 3) | 10 | 0.4 |

**S2 Table.** Number and proportion of samples that could not be haplotyped at different steps of the haplotyping and sub-haplotyping. In brackets are given the tables that are referred to in the manuscript.
