# Supplementary material for: Complex spatio-temporal distribution and genomic ancestry of mitochondrial DNA haplogroups in 24,216 Danes
Source: PLoS One. 2018 Dec 13;13(12):e0208829. doi: 10.1371/journal.pone.0208829 (PMC6292624; doi:10.1371/journal.pone.0208829)
Supplement: S3 Table — U*: All U hgs not belonging to any of the specified U sub-hgs. (DOCX) [file pone.0208829.s008.docx]

**S3 Table.** Proportion of different Hg-U subhaplogroups in 1981-1986 and from 2000-2005. U*: All U hgs not belonging to any of the specified U sub-hgs.

| Haplogroup | 1981-1986 | 2000-2005 | P-Value |
| --- | --- | --- | --- |
|  | **% (n)** | **% (n)** |  |
| U* | 4.6 (26) | 4.1 (32) | 0.68 |
| U1 | 1.6 (9) | 2.3 (18) | 0.43 |
| U2-U3 | 19.5 (111) | 19.1 (150) | 0.83 |
| U4-9 | 20.1 (114) | 19.7 (155) | 0.89 |
| U5a | 34.9 (198) | 31.6 (249) | 0.22 |
| U5b | 14.8 (84) | 15.6 (123) | 0.70 |
| U6 | 0.2 (1) | 1.0 (8) | 0.09 |
| U7 | 2.3 (13) | 3.3 (26) | 0.32 |
| U8 | 2.1 (12) | 3.3 (26) | 0.24 |
| Total | 100 (568) | 100 (787) | - |
