# Supplementary material for: Complex spatio-temporal distribution and genomic ancestry of mitochondrial DNA haplogroups in 24,216 Danes
Source: PLoS One. 2018 Dec 13;13(12):e0208829. doi: 10.1371/journal.pone.0208829 (PMC6292624; doi:10.1371/journal.pone.0208829)
Supplement: S4 Table — (DOCX) [file pone.0208829.s009.docx]

**S4 Table.** Relative proportions of macro-hgs L, M and U in metropolitan and rural areas in 1981-1986 and 2000-2005.

| Haplogroup | Area | 1981-1986 % (n) | 2000-2005 % (n) |
| --- | --- | --- | --- |
| L | Metropolitan | 0.6(5) | 2.2(29) |
|  | Rural | 0.1(3) | 0.9(38) |
| M | Metropolitan | 2.4(20) | 3.4(45) |
|  | Rural | 0.6(24) | 2(86) |
| U | Metropolitan | 14.1(116) | 13.6(178) |
|  | Rural | 12.3(463) | 14.5(612) |
